# Supplementary material for: Clinical Severity of SARS-CoV-2 Omicron Variant Compared with Delta among Hospitalized COVID-19 Patients in Belgium during Autumn and Winter Season 2021–2022
Source: Viruses. 2022 Jun 14;14(6):1297. doi: 10.3390/v14061297 (PMC9227815; doi:10.3390/v14061297)
Supplement: Supplementary file 1 [file viruses-14-01297-s001.zip › viruses-1740737-supplementary.pdf]

Table S1. Standardized risk per variant (in %), Relative Risk (RR) and Risk Difference (RD, in %) estimates and 95% Confidence Interval (CI) for severity outcomes within a multi-center cohort study to assess the impact of SARS-CoV-2 variants on COVID-19 disease severity among hospitalized patients without a documented previous infection in Belgium. This sensitivity analysis was based on a subgroup of 941 hospitalized patients (433 patients infected with the Omicron variant and 508 patients infected with the Delta variant).

Outcome	Standardized Risk [95% CI] in %		RR [95% CI]	RD [95% CI]
	Omicron	Delta		
Severe COVID-19 [1]	22.5 [12.1; 32.8]	35.2 [26.5; 44.0]	0.64 [0.30; 0.98]	-12.8 [-26.8; 1.3]
ICU admission	12.4 [3.0; 21.8]	21.7 [17.6; 25.9]	0.57 [0.11; 1.03]	-9.4 [-19.2; 0.5]
In-hospital mortality	19.1 [9.3; 29.0]	24.1 [16.2; 32.1]	0.79 [0.30; 1.29]	-5.0 [-17.3; 7.3]

[1] Defined as either intensive care unit (ICU) admission, and/or acute respiratory distress syndrome (ARDS), and/or in-hospital mortality.

Table S2. Standardized risk per variant (in %), Relative Risk (RR) and Risk Difference (RD, in %) estimates and 95% Confidence Interval (CI) for severity outcomes within a multi-center cohort study to assess the impact of SARS-CoV-2 variants on COVID-19 disease severity among hospitalized patients with a confirmed (through whole genome sequencing) Omicron or Delta infection in Belgium. This sensitivity analysis was based on a subgroup of 619 hospitalized patients (251 patients infected with the Omicron variant and 368 patients infected with the Delta variant).

Outcome	Standardized Risk [95% CI] in %		RR [95% CI]	RD [95% CI]
	Omicron	Delta		
Severe COVID-19 [1]	25.9 [13.0; 38.8]	39.3 [30.5; 48.1]	0.66 [0.29; 1.03]	-13.4 [-29.0; 2.2]
ICU admission	11.2 [0.0; 23.7]	25.0 [20.0; 29.9]	0.45 [0.00; 0.97]	-13.8 [-26.6; -1.0]
In-hospital mortality	24.6 [12.6; 36.7]	28.4 [18.0; 38.9]	0.87 [0.00; 2.31]	-3.8 [-22.2; 14.5]

[1] Defined as either intensive care unit (ICU) admission, and/or acute respiratory distress syndrome (ARDS), and/or in-hospital mortality.

Table S3. Standardized risk per variant (in %), Relative Risk (RR) and Risk Difference (RD, in %) estimates and 95% Confidence Interval (CI) for severity outcomes within a multi-center cohort study to assess the impact of SARS-CoV-2 variants on COVID-19 disease severity among hospitalized patients in Belgium when only considering variant information obtained through baseline genomic surveillance. This sensitivity analysis was based on a subgroup of 448 hospitalized patients (183 patients infected with the Omicron variant and 265 patients infected with the Delta variant).

Outcome	Standardized Risk [95% CI] in %		RR [95% CI]	RD [95% CI]
	Omicron	Delta		
Severe COVID-19 [1]	26.4 [10.2; 42.6]	31.2 [24.7; 37.7]	0.85 [0.26; 1.43]	-4.8 [-22.2; 12.6]
ICU admission	8.3 [0.0; 23.7]	23.6 [16.2; 31.1]	0.35 [0.00; 1.12]	-15.3 [-32.2; 1.5]
In-hospital mortality	25.4 [7.7; 43.1]	21.5 [11.0; 32.0]	1.18 [0.00; 3.29]	3.9 [-20.7; 28.5]

[1] Defined as either intensive care unit (ICU) admission, and/or acute respiratory distress syndrome (ARDS), and/or in-hospital mortality.
